# IRVING S. WRIGHT AWARD, VINCENT CRISTOFALO AWARD, AND TERRIE FOX WETLE AWARD PRESENTATIONS AND LECTURES

**DOI:** 10.1093/geroni/igac059.647

**Published:** 2022-12-20

**Authors:** James Kirkland

## Abstract

The Irving S. Wright Award of Distinction Lecture will feature an address by the 2022 recipient Thomas M. Gill, MD of Yale University. The Vincent Cristofalo Rising Star Award in Aging Research lecture will feature an address by the 2022 recipient Jamie Nicole Justice, PhD, of Wake Forest University. The Terrie Fox Wetle Award lecture will feature an address by the 2022 recipient Benjamin H. Han, MD, MPH, of the University of California San Diego. These awards are given by the American Federation for Aging Research, Inc.

